# Lifting of Social Distancing Measures: Perspectives From Vietnam

**DOI:** 10.1017/dmp.2020.238

**Published:** 2020-07-14

**Authors:** Trang H.D. Nguyen

**Affiliations:** Institute of Biotechnology and Food Technology, Industrial University of Ho Chi Minh City, Ho Chi Minh City, Vietnam

**Keywords:** COVID-19, reopening, social distancing, socio-economic development, Vietnam

## Abstract

While many nations are struggling to slow the transmission rate of the coronavirus disease 2019 (COVID-19), Vietnam has seen no new locally acquired cases since April 16. After implementing 22 d of nationwide social distancing, on April 23, the government of Vietnam announced the easing of social distancing measures. This allows the country to restart its socio-economic activities in a gradual, prudent manner. Domestic tourism and exports of agricultural and anti-COVID-19 medical products take priority over the other sectors in this postpandemic economic recovery. Importantly, the country needs to stay vigilant on the fight against the disease to prevent a possibility of another outbreak.

After imposing 15 d of nationwide social distancing and a “stay-at-home” order from April 1, 2020, to contain community transmission of the new coronavirus, Vietnam started seeing no daily new cases within its borders. The country announced a 1-wk extension to the social distancing to ensure that local transmission was completely under control. On April 23, the country’s government eased social distancing guidelines for almost all provinces and cities. This report is to share the country’s roadmap to loosening social distancing restrictions so as to effectively controlling the COVID-19 situation and ensuring socio-economic development.

## REPORT

### Lifting of Social Distancing

**FIGURE 1:**
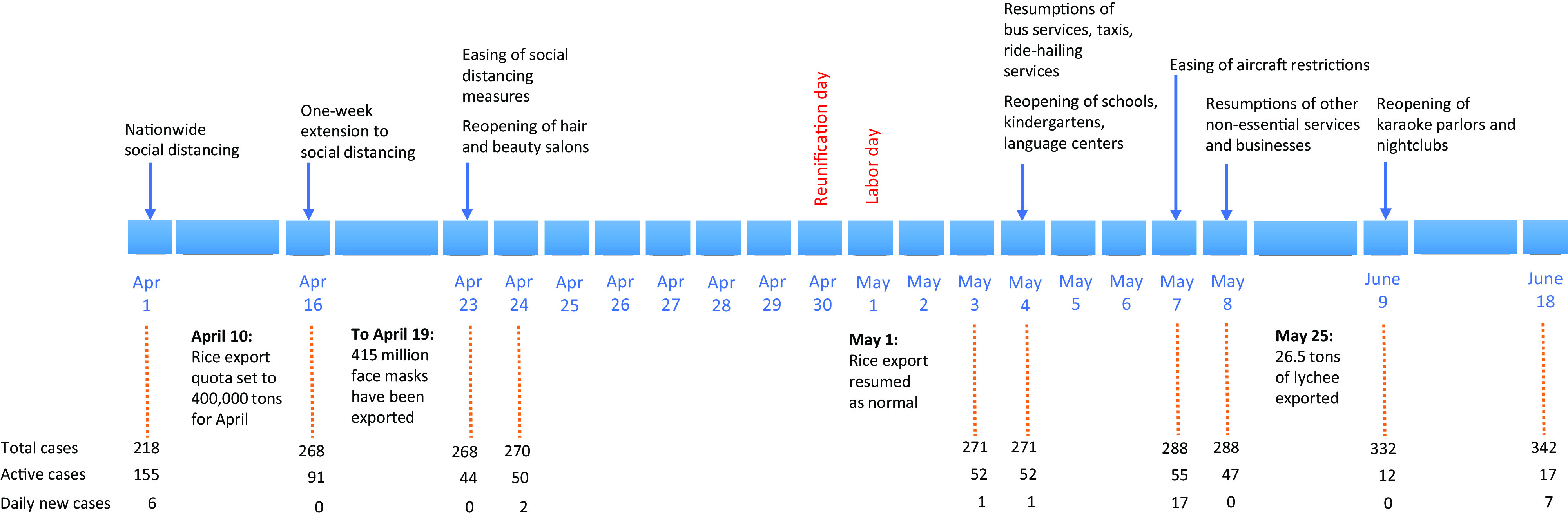
Timeline of Lifting of Social Distancing Measures in Vietnam. As of April 16, daily new cases denote overseas-acquired cases

While most of the localities across the country are no longer classified as high-risk areas, specific districts, such as Me Linh and Thuong Tin in Hanoi, the capital of Vietnam, still remained on lockdown until May 14. The extension of lockdown was due to the last COVID-19 patients in these districts reported on April 15. Under the new guidelines, schools and kindergartens reopened on May 4 or 1 wk later, depending on each locality’s situation. The normal operation of public and inter-provincial bus services, traditional taxis, and ride-hailing services has resumed since May 4. On May 7, the country loosened social distancing restrictions on aircraft and removed limitations on the number of passengers, facilitating repatriation of thousands of Vietnamese nationals stranded abroad.^1^ Bans on foreigners from entering Vietnam continue to be effective, with exceptions for foreigners on diplomatic, official business, and skilled workers. While hair and beauty salons reopened as early as on April 23, other nonessential services and businesses, such as restaurants, gyms, theaters, religious gatherings, and public gatherings of more than 30 people, continued to be suspended until May 8. On June 9, the Prime Minister of Vietnam agreed to resume karaoke parlors and nightclubs, although the specific reopening date will depend on each locality (Figure 1).

### Resumption of Exports of Agricultural and Medical Products

The country’s rice export sector has been adversely affected by the pandemic. As the world’s third largest rice exporter, Vietnam ordered a temporary suspension of rice exports while reviewing domestic inventories on March 24. It resumed rice exports with a quota raised to 400,000 metric tons for April, and subsequently allows normal rice exports from May 1. In addition, the country has exported the first batch of 26.5 tons of lychee in the 2020 season to Singapore, Australia, and the United States.^2^

There is a global shortage of medical face masks amidst the pandemic while domestic demand is met. Therefore, in early April, the country’s Prime Minister approved exports of medical products, such as face masks and protective clothing, to nations hardest hit by the pandemic. Thus far, it has exported approximately 415 million medical face masks to Japan, Korea, Germany, and the United States.^3^

### Health-care System

In the battle against COVID-19, the government of Vietnam’s affordable and effective approach is to convert army camps, college and university dormitories, and hotels/resorts into centralized quarantine zones. These facilities are used for isolating suspected COVID-19 cases, repatriates, and foreign visitors to the country. To prevent and control the spread of the disease in the community, Vietnam has endeavored to test anyone that may have had direct contact with infection sources. For example, if there is a confirmed case linked to local transmission, the patient must immediately undergo treatment at a provincial hospital while all direct contacts, referred to as F1, are required to test for COVID-19 and are sent to a quarantine zone. Those, who have close contact with F1 need to be self-isolated.

Currently, more than 110 laboratories across the country are able to conduct real-time polymerase chain reaction (PCR) tests for detection of the new coronavirus, with a total capacity of 27,000 samples per day. As of April 30, approximately 261,000 tests had been performed, with 2684 tests per million people.^4^

### Current Situation

As of June 18, the country has documented 342 positive cases of COVID-19, with 325 of these having recovered. And though there have been 74 new infected cases over the past 60 d, these are Vietnamese returnees mostly from the United Arab Emirates and Russia, and were quarantined upon arrival. No new locally acquired cases have been recorded in the country since April 16. Hence, it ponders an end of the pandemic in the country. Nevertheless, the country still needs to encourage its citizens to use face masks, frequently wash hand, stay vigilant, and avoid complacency.

## DISCUSSION

Notably, Vietnam prudently allowed the normal operation of public transportation and taxi services to resume in the week following the country’s National Reunification and International Labor Days (ie, April 30 and May 1). The explanation for this resumption is to help prevent possible massive passenger transport and public gatherings during the holidays, minimizing potential risks of spreading COVID-19.^5^

Vietnam is among the countries that swiftly imposed strict travel restrictions in response to the pandemic. Despite hammering the tourism industry, the travel restrictions have shown effectiveness in preventing travel-related infection cases and local spread of the disease. Recently, early detection and quarantine have been recognized as being more effective in alleviating this pandemic than travel restrictions.^6^ The country requires that all people entering the country by means of air, sea, or land undergo medical checks and 14-d quarantine at government-run facilities upon arrival to effectively contain local transmission of the new coronavirus.^7,8^ Unlike repatriation by means of air, returnees by means of land borders, particularly Cambodia, may not always enter the country legally,^9^ making it difficult to contain COVID-19. To prevent and control this situation, local authorities need to work in coordination with local people and relatives of illegal returnees.

Vietnam’s tourism sector, which plays an important role in the country’s economic development, appears to be hard hit by the pandemic. Fear and anxiety about the disease at the early stage of the COVID-19 outbreak in January caused tourism to plummet in Vietnam. It is estimated that the number of domestic travelers and foreign arrivals to the country decreased by 6% and 18%, respectively.^10^ The easing of social distancing measures has set the stage for the return of domestic tourism in Vietnam. The Vietnam Ministry of Culture, Sports, and Tourism in cooperation with localities, airlines, transport, and travel agencies implemented the “Safe Vietnam Tourism” campaign and a stimulus package for domestic tourism. The country is also expected to replicate its resumption of domestic tourism by focusing on neighboring Asian markets, such as China and Korea. A very recent survey of 1000 qualified Chinese travelers has indicated that approximately 45% of the respondents favor tours in Vietnam for post–COVID-19 travel.^11^ However, it is prudent to not currently reopen doors to foreign tourists, as the COVID-19 situation in the world is rapidly evolving and can be hardly foreseeable, hinting at the high risk of another outbreak.

## CONCLUSIONS

In summary, while many parts of the world are still on lockdown amidst the pandemic, Vietnam has lifted social distancing restrictions and is rebooting its socio-economic activities. These need to be conducted in a step-by-step manner that suits each locality’s situation. Meanwhile, exports of agricultural and anti-COVID-19 medical products and promotion of domestic tourism are prioritized. Importantly, the country needs to implement measures to closely control and monitor repatriation and immigration by means of its borders.
